# Eliminating malaria by 2040 among agricultural households in Africa: potential impact on health, labor productivity, education and gender equality

**DOI:** 10.12688/gatesopenres.12843.2

**Published:** 2018-11-09

**Authors:** Derek W. Willis, Nick Hamon

**Affiliations:** 1 Center for Research on Environmental Decisions, Columbia University, New York, NY, 10027, USA; 2 IVCC, Pembroke Place, Liverpool, L3 5QA, UK

**Keywords:** malaria eradication, agricultural households, Africa, sustainable development goals

## Abstract

**Background**: Ambitious goals have been set to eradicate malaria by the year 2040. Given the high poverty levels and the intense levels of malaria transmission in sub-Saharan Africa, suppressing malaria in rural agricultural communities in these regions will be one of the greatest challenges to achieving malaria eradication. This study has two objectives. The first is to estimate how eradicating malaria by 2040 would affect agricultural households in sub-Saharan Africa. The second is to identify where additional research is needed to develop better estimates of how eradicating malaria by 2040 would affect those households.

**Methods**: Using agricultural census data and malaria morbidity data, we developed estimates of the number of malaria cases in 2018 among agricultural households with fewer than 10 hectares of land for 35 countries in sub-Saharan Africa. By combining these estimates with additional evidence from the literature, we analyzed how achieving malaria eradication by 2040 would affect indicators related to four Sustainable Development Goals: health, poverty, education and gender equality.

**Results**: Our analysis found that achieving malaria eradication by 2040 would prevent approximately 841 million cases of malaria and thereby decrease the number of lost workdays among agricultural households by approximately 3.2 billion days. Eradicating malaria by 2040 would also increase the number of school days attended by children by 1.5 billion days while also reducing the number of caregiving days provided by women for malaria cases by approximately 1.1 billion days.

**Conclusions**: This article analyzes the impact of eradicating malaria among agricultural households in sub-Saharan Africa using indicators related to four of the Sustainable Development Goals. Enhanced data collection efforts related to these four indicators would facilitate more rigorous estimates of how eradicating malaria would affect these indicators over the next two decades.

## Introduction

An ambitious goal has been set to eradicate malaria by the year 2040 ( Gates, n.d.). Given the high poverty levels and the intense levels of malaria transmission in sub-Saharan Africa, suppressing malaria in rural agricultural communities in these regions will be one of the greatest challenges to achieving worldwide malaria eradication ( Kiszewski *et al*., 2004). Achieving malaria eradication in these communities would not only be a significant public health milestone but could also decrease poverty, increase levels of childhood education and improve gender equality.

The Sustainable Development Goals (SDGs) provide a framework for understanding how suppressing malaria would affect health, poverty, education, and gender equality among agricultural households in sub-Saharan Africa ( United Nations, 2015). An increased understanding of how eradicating malaria would affect these households could increase interest in collaborative efforts between the public health and agricultural sectors.

This study has two objectives. The first objective is to estimate how eradicating malaria by 2040 would affect the health, poverty, education and gender equality of agricultural households in sub-Saharan Africa. The second is to identify where additional research is needed to develop better estimates of how eradicating malaria would affect these households.

Using agricultural census data and malaria morbidity data, we developed estimates of the number of malaria cases among agricultural households for 35 countries in sub-Saharan Africa ( Willis, 2018). Using these estimates, we analyzed two paths for the malaria burden among agricultural household from 2018 through 2040. The first path, the Status Quo Path, assumes that the annual malaria morbidity burden among agricultural households remains at its current level through 2040. The Malaria Elimination Path assumes that malaria cases among these households will decrease annually from 2018 levels to nil in 2040. For each path, we estimated annual indicators related to the following SDGs: health, poverty, education and gender equality. We estimate the impact of eradicating malaria by 2040 on these indicators by comparing the same indicators for the Status Quo Path and Malaria Elimination Path.

## Methods

### Status Quo Path versus Elimination Path

Our methodology enables us to compare our selected indicators for the SDGs for the Status Quo Path and Malaria Elimination Path. The Malaria Elimination Path refers to the indicators that would occur from 2018 through 2040 in each country if the annual number of malaria cases decreased from 2018 levels to nil in 2040. The Status Quo Path is the counterfactual case for the corresponding indicators if the number of malaria cases in each country remained at their 2018 levels through 2040.

**Figure 1.**
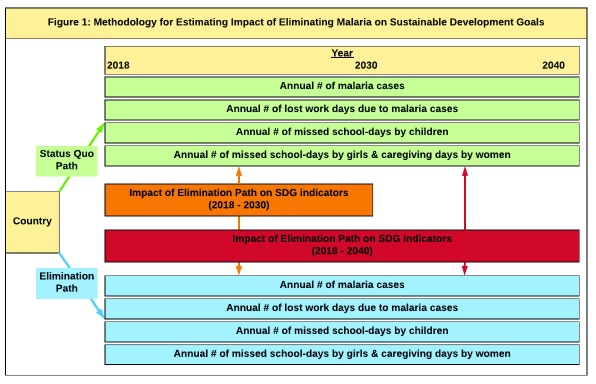
Methodology for estimating impact of eliminating malaria on Sustainable Development Goals.

We compare the selected SDG indicators from the Status Quo Path and Malaria Elimination Path for two overlapping time periods, 2018 to 2030 and 2018 to 2040. The relevant SDGs are summarized in Table 1. The SDG indicators that we estimated using our dataset are described in Table 2, and Figure 1 summarizes our methodology.

**Table 1.** Relevant Sustainable Development Goals for evaluating potential impact of suppressing malaria among agricultural households in Sub-Saharan Africa. Source: ‘Sustainable Development Goals - 17 Goals to Transform Our World’ ( United Nations, 2015).

Goal	Target for 2030
**#1: No poverty**	“By 2030, eradicate extreme poverty for all people everywhere, measured as people living on less than $1.90 a day” ( United Nations).
**#3: Good health and** **well-being**	“By 2030, reduce the global maternal mortality ratio to less than 70 per 100,000 live births” ( United Nations).
	“By 2030, end preventable deaths of newborns and children under 5 years of age, with all countries aiming to reduce neonatal mortality to at least as low as 12 per 1,000 live births and under-5 mortality to at least as low as 25 per 1,000 live births” ( United Nations).
**#4: Quality education**	“By 2030, ensure that all girls and boys complete free, equitable and quality primary and secondary education leading to relevant and Goal-4 effective learning outcomes” ( United Nations).
**#5: Gender equality**	“Adopt and strengthen sound policies and enforceable legislation for the promotion of gender equality and the empowerment of all women and girls at all levels” ( United Nations).

**Table 2.** Indicators for evaluating impact of suppressing malaria among agricultural households on the relevant Sustainable Development Goals.

Goal	Indicator (only agricultural households in Sub-Saharan Africa)
**#1: No poverty**	Lost work days from malaria cases among agricultural households
**#3: Good health and well-being**	Malaria cases among agricultural households
**#4: Quality education**	No. of lost school days by all children due to malaria cases among agricultural households
**#5: Gender equality**	No. of lost school days by girls due to malaria cases among agricultural households
	No. of caregiving days by women due to malaria cases among agricultural households

The first time period, from 2018 to 2030, corresponds to the target year of 2030 for achieving the SDGs; 2030 is also the final year of the “Global Technical Strategy for Malaria” ( World Health Organization, 2015). This strategy has established a goal of suppressing the incidence of malaria cases by 90 percent by 2030 relative to 2015 levels ( World Health Organization, 2015). The second period, from 2018 to 2040, corresponds to the goal of eradicating malaria by 2040 ( Malaria No More, 2015).

### Three scenarios: most conservative, base case and least conservative

A variety of different values could be used to develop and analyze our dataset in order to estimate the impact of eliminating malaria on the SDG indicators. Given this uncertainty, we use three different scenarios for developing and analyzing our dataset: the most conservative scenario, the base case scenario, and the least conservative scenario.

Our most conservative scenario represents the parameter estimates associated with a lower impact of malaria eradication on the indicators. The least conservative scenario is our set of parameter values associated with the largest impact of eradicating malaria on the indicators. An intermediate set of parameter values are modeled in our base case scenario.

## Detailed summary of methodology

Here, we describe each step for developing and analyzing our dataset in more detail.

### Definition of smallholder agricultural households

We will define agricultural households using the same standards that have been used in many agricultural censuses of countries in sub-Saharan Africa. The following definition in Ethiopia’s agricultural census in 2010 is representative of the definitions used in most agricultural censuses conducted in Africa:

• A household is considered an agricultural household when at least one member of the household is engaged in growing crops and/or raising livestock in private or in combination with others ( Federal Democratic Republic, 2010/2011).

### Estimates of the population of agricultural households in sub-Saharan Africa in 2018

Our analysis included only countries in sub-Saharan Africa in which more than half of the country experienced malaria transmission. We therefore excluded Namibia and South Africa from our analysis and focused on the following countries: Angola, Benin, Botswana, Burkina Faso, Burundi, Cameroon, Central African Republic, Chad, Republic of Congo, Democratic Republic of Congo, Equatorial Guinea, Ethiopia, Gabon, Gambia, Ghana, Guinea, Guinea Bissau, Côte d'Ivoire, Kenya, Liberia, Madagascar, Malawi, Mali, Mozambique, Niger, Nigeria, Rwanda, Senegal, Sierra Leone, South Sudan, Tanzania, Togo, Uganda, Zambia and Zimbabwe.

We used several steps to develop our estimates of the number of agricultural households in each country in 2018 because these data are not available from existing datasets.

First, we developed estimates of the total number of agricultural households in 2018 for each country using a study published in 2016 that includes agricultural census data from most countries in sub-Saharan Africa ( Lowder *et al*., 2016). Using these agricultural census data, we estimated that there are approximately 72.8 million agricultural households in the 35 target countries. Agricultural census data were not available for any year for Equatorial Guinea and South Sudan. We estimated the total number of agricultural households in Equatorial Guinea and South Sudan for 2018 using the proportion of agricultural households relative to the total population in neighboring countries.

The next step in developing our dataset was estimating the number of agricultural households with agricultural areas of fewer than 10 hectares. We assumed that households with agricultural areas of fewer than 10 hectares would be more directly affected by malaria, as larger farms would be more likely to rely on labor hired from outside the household.

We used a supplementary dataset developed by Lowder *et al.* to develop our estimates of the number of households with agricultural areas of fewer than 10 hectares in malarious regions of sub-Saharan Africa ( Lowder *et al*., 2016). Data for the agricultural area held by households of more than 10 hectares of land were available only for the following countries: Burkina Faso, Ivory Coast, Ethiopia and Uganda. For each of these countries, we calculated the proportion of the total agricultural area (agricultural area of all agricultural households) that was held by agricultural households with fewer than 10 hectares of land. For example, the total agricultural area held by all agricultural households in Uganda, according to a census conducted in 1991, was 3,668,288 hectares. Of that total, approximately 2,570,401 hectares were held by agricultural households that held fewer than 10 hectares each. Therefore, approximately 70% of the total agricultural area that was held by agricultural households in Uganda in 1991 was held by households with less than 10 hectares. The average of this proportion across all four countries (Burkina Faso, Ivory Coast, Ethiopia and Uganda) was 74%. We assumed that 74% of all agricultural households in each of the remaining countries in our dataset held less than 10 hectares of land for farming.

We would expect that these estimates for the number of agricultural households with fewer than 10 hectares of land in malarious regions of sub-Saharan Africa represent lower-bound estimates, as some of the estimates rely on agricultural census data from the 1980s and 1990s.

### Annual estimates of total population in agricultural households per country from 2018 to 2040

Next, we developed annual estimates of the population of agricultural households with less than 10 hectares in each of the targeted countries from 2018 through 2040, as these population estimates were not available in the literature.

We assumed that the number of agricultural households in 2018 remained the same through 2040. Although we would expect the overall population growth rate of sub-Saharan African countries to be around 2.16% through 2040 ( World Bank, 2018), the population in rural areas of Africa is not expected to grow at the same rate ( 2018 Revision of World Urbanization Prospects, 2018). By contrast, sub-Saharan Africa will experience high urban population growth through 2040:

• Sub-Saharan Africa (SSA) is often regarded as the world’s fastest urbanizing region. Urban areas currently contain 472 million people, and will double over the next 25 years. The global share of African urban residents is projected to grow from 11.3 percent in 2010 to 20.2 percent by 2050 ( Saghir, 2018).

We assumed that, on average, six individuals reside in each agricultural household. This estimate was based on the number of individuals per household in a recent study of malaria’s impact on harvest values in Zambia ( Fink & Masiye, 2015). Estimates of the annual population of agricultural households with less than 10 hectares for each country are calculated by multiplying the annual number of agricultural households of that size by six.

### Annual estimates of number of malaria cases among agricultural households per country from 2018 to 2040 - Status Quo Path

We use the term Status Quo Path to refer to the number of malaria cases in each sub-Saharan African country from 2018 through 2040 if the anti-malaria programs currently being implemented in those countries remain unchanged.

We use three different approaches (most conservative scenario, base case scenario, least conservative scenario) for estimating the Status Quo Path for the annual number of malaria cases among agricultural households per country from 2018 through 2040.

***Most conservative scenario.*** For our most conservative scenario, we estimated the annual number of malaria cases among agricultural households with less than 10 hectares per country by using the average of the total number of malaria cases per country from 2012 through 2016 as reported in the World Malaria Report for 2017 ( WHO, 2017). We divided the average of the total annual number of malaria cases in a country from 2012 through 2016 with the country’s total population in 2016 to calculate the number of malaria cases per person in the country. We assumed that the estimate of the number of malaria cases per person in 2016 in the country is the same as the number of malaria cases per person in 2018. By multiplying the number of malaria cases per person in 2018 by the total population of agricultural households with less than 10 hectares in 2018, we estimated the number of malaria cases among agricultural households of that type in 2018.

For the Status Quo Path, we assumed that the number of malaria cases among these households in 2018 remained unchanged for the subsequent years through 2040. This approach to estimating the number of malaria cases among agricultural households assumed that the malaria risk experienced by agricultural households is the same as the malaria risk of non-agricultural households.

Our approach for estimating the number of malaria cases among agricultural households was conservative for two reasons and will therefore likely underestimate the actual annual number of malaria cases in each country. First, agricultural households will be primarily in rural areas and the malaria risk for households in rural areas will likely be significantly greater than in urban areas. Therefore, the assumption that malaria risk is the same among all households will certainly underestimate the number of malaria cases experienced by households in rural areas. Second, a significant proportion of malaria cases that occur in any given country are not recorded in a country’s official annual estimates. A recent study estimated that of the approximately 252 million malaria cases in sub-Saharan Africa that could have been detected with active case detection, only 34% would be recorded through the use of passive case detection ( Griffin *et al.*, 2014).

***Base case scenario.*** To account for the potential number of malaria cases that are unreported, our base case scenario assumes that approximately one-third of the actual number of malaria cases that occur among agricultural households are unreported. We therefore multiply the annual number of malaria cases estimated under the most conservative scenario by 1.5 to derive annual estimates for each country for this scenario.

***Least conservative scenario.*** For estimates of the annual number of malaria cases under our least conservative scenario, we assumed that approximately half of the actual number of malaria cases that occur are reported. Accordingly, we multiply the annual number of malaria cases from the most conservative scenario by 2 in order to estimate the annual number of malaria cases for the least conservative scenario. This approach of multiplying the annual number of malaria cases by 2 is justified based on a study that estimated that approximately only one-third of cases are recorded through passive case detection ( Griffin *et al.*, 2014).

### Annual estimates of number of malaria cases among agricultural households per country from 2018 to 2040 - Malaria Elimination Path

For the Malaria Elimination Path, we used the same estimates for the number of malaria cases in 2018 for each scenario from the Status Quo Path. However, we assumed the annual number of malaria cases for each scenario decreased to zero in 2040.

### Annual estimates of number of lost work days due to malaria cases among agricultural households per country from 2018 to 2040 - Status Quo Path and Malaria Elimination Path

To estimate the potential impact of eliminating malaria among agricultural households on poverty we used the number of lost work days due to malaria cases as an indicator. We used two steps to estimate the number of work days lost due to malaria cases.

First, we estimated the proportion of malaria cases that occurred annually among children and adults. We defined children as age 15 years or younger. Adults were defined as being older than 15 years. An analysis of the age distribution of malaria cases due to *Plasmodium falciparum* found that approximately 48% of malaria cases in 2010 occurred in children under the age of 5, while between 20% and 40% of all cases occurred in children age 5 to 15 ( Griffin *et al*., 2014). We therefore assumed that 70% of all malaria cases occurred in children under the age of 5 and 30% occurred in children over that age.

Next, we developed estimates of the number of work days lost for malaria cases among adults and children. We defined lost work days as days lost by an adult who experiences a case of malaria and days lost by an adult when providing care for a child with malaria. Studies of the number of lost work days due to a malaria case among adults in an agricultural household yield a wide range of values. Most studies estimated the number of lost work days by adults in sub-Saharan Africa due to malaria as ranging from 3 to 7 days ( Asenso-Okyere & Dzator, 1997; Attanayake *et al*., 2000; Gazin *et al*., 1988; Guiguemdé *et al*., 1997; Larochelle & Dalton, 2005; Leighton & Foster, 1993; Nur & Mahran, 1988; Organization & Espd, 2005; Sauerborn *et al*., 1991). Studies of the number of adult-provided caregiving days for malaria cases in children put the number between 1 and 5 days ( Asenso-Okyere & Dzator, 1997; Ettling *et al*., 1994; Leighton & Foster, 1993; Nur, 1993). We used values within these ranges in each of our scenarios to develop estimates of the total number of lost work days among agricultural households due to malaria.

For our analysis using our most conservative scenario, we assumed that a malaria case in an adult led to a loss of 3 work days, while a malaria case in a child resulted in the loss of 1 adult work day. Our base case scenario assumed that 5 adult work days were lost from malaria and 3 adult work days were lost to care for a child. Finally, our least conservative scenario assumed 7 work days were lost per adult malaria case and caring for a child led to a loss of 5 work days.

### Annual estimates of missed school days due to malaria cases among agricultural households per country from 2018 to 2040 - Status Quo Path and Malaria Elimination Path

Our indicator for the impact of malaria cases on the SDG of improving education was the number of school days missed due to malaria. Most studies estimated that the number of missed school days per malaria case varied from 4 to 8 days ( Brooker *et al*., 2000; Bundy *et al*., 2000; Chima *et al*., 2003; Chuma *et al*., 2010; Konradsen *et al*., 1997; Leighton & Foster, 1993; Sachs & Malaney, 2002). Our most conservative scenario assumed a child missed 4 school days per malaria case while our least conservative scenario assumed 8 school days were missed per case. The base case analysis used an estimate of 6 missed school days per malaria case.

### Annual estimates of missed school days by girls due to malaria cases and caregiving days by women for malaria cases

The two indicators we used to estimate malaria’s impact on gender equality are missed school days by girls due to malaria and the number of caregiving days by women for malaria cases among children in their household. We estimated the number of malaria cases among girls by assuming that half of all malaria cases among children are experienced by girls. We used the same number of missed school days per malaria case for each scenario as described for the previous indicator.

Limited data are available regarding the proportion of caregiving days provided by women for malaria cases experienced by children in a household. However, it is safe to assume that women provide the vast majority of caregiving and we therefore assumed that women provide between 60% and 90% of all caregiving days for malaria cases experienced by children in an agricultural household. Our most conservative scenario assumed that women provided 60% of all caregiving for malaria cases experienced by children. We assumed for our base case scenario that 75% of all caregiving was provided by women and assumed 90% for our least conservative scenario.

## Summary of parameter values for each scenario

The parameter values used in each scenario are summarized in Table 3.

**Table 3.** Summary of parameter values for each scenario.

Parameter	Most conservative scenario	Intermediate Scenario	Least conservative scenario
Malaria cases among agric. HHs for 2018	(Total No. malaria cases per country / total population per country) × pop. of agric. HHs	1.5 × most conservative scenario	2 × most conservative scenario
No. lost work days due to caregiving for child cases	1	3	5
No. lost work days due to adult cases	3	5	7
No. lost school days for school- age child cases	4	6	8
% of caregiving days provided by women for each child case	60%	75%	90%

## Results

We analyzed the dataset we developed ( Willis, 2018) in order to determine how eliminating malaria among agricultural households in sub-Saharan Africa by 2040 would potentially affect indicators related to four of the SDGs. As described in the Methods section, our analysis focused on the following five indicators for agricultural households in 35 countries in sub-Saharan Africa: number of malaria cases, number of lost work days due to malaria, number of lost school days by children due to malaria, number of lost school days by girls due to malaria and number of caregiving days provided by women for cases of malaria.

### Population of agricultural households in sub-Saharan Africa

Our analysis of the agricultural census data for the countries included in our study found that there were approximately 54 million agricultural households in 2018 with farming areas of less than 10 hectares out of a total number of approximately 73 million agricultural households. Based on our estimate of 6 people per household, the total population of agricultural households with less than 10 hectares in 2018 was approximately 324 million.

### 2018 to 2030: Impact of Malaria Elimination Path on SDGs

Table 4 summarizes the impact of suppressing malaria among agricultural households from 2018 to 2030. We analyzed this time period because 2030 is the target year for achieving the SDGs.

**Table 4.** Impact of malaria elimination path on Sustainable Development Goals (2018 to 2030).

		Most Conservative Scenario (2018 to 2030)			Base Case Scenario (2018 to 2030)			Least Conservative Scenario (2018 to 2030)		
Sustainable Development Goal	Indicator	Status Quo	Elimination Path	Impact by 2030	Status Quo	Elimination Path	Impact by 2030	Status Quo	Elimination Path	Impact by 2030
#1: No Poverty	# of lost work days from malaria cases among agric. HHs	1,021,564,208	742,955,788	-278,608,420	3,447,779,203	2,507,475,784	-940,303,419	7,150,949,458	5,200,690,515	-1,950,258,943
#3: Good Health & Well- being	# of malaria cases among agric. HHs	638,477,630	464,347,367	-174,130,263	957,716,445	696,521,051	-261,195,394	1,276,955,260	928,694,735	-348,260,526
#4: Quality Education	# of lost school days by children due to malaria cases among agric. HHs	766,173,156	557,216,841	-208,956,315	1,723,889,602	1,253,737,892	-470,151,710	3,064,692,625	2,228,867,364	-835,825,261
#5: Gender equality	# of lost school days by girls due to malaria cases among agric. HHs	383,086,578	278,608,420	-104,478,158	861,944,801	626,868,946	-235,075,855	1,532,346,312	1,114,433,682	-417,912,631
#5: Gender equality	# of caregiving days by women due to malaria cases among agric. HHs	268,160,605	195,025,894	-73,134,710	1,508,403,401	1,097,020,656	-411,382,746	4,022,409,070	2,925,388,415	-1,097,020,656

HH, household.

***Malaria cases among agricultural households.*** Under our base case scenario, there would be approximately 697 million malaria cases among agricultural households from 2018 through 2030 if malaria elimination were achieved in 2040. By contrast, approximately 958 million malaria cases would occur over that same period if the status quo were maintained. As a result, approximately 261 million malaria cases would be prevented from 2018 through 2030 by pursuing the path to eradication by 2040. A lower bound for the number of malaria cases prevented by pursuing eradication can be estimated with the most conservative scenario (174 million), while an upper bound is provided by the least conservative scenario (348 million).

***Work days among agricultural households.*** The base case scenario estimate for the number of additional work days gained by suppressing malaria from 2018 to 2030 is approximately 940 million days. The lower bound for this estimate is 279 million work days from our most conservative scenario analysis and the upper-bound estimate is 1.95 billion work days.

***School days among children in agricultural households.*** Approximately 470 million additional school days by children of agricultural households would be gained if malaria were suppressed between 2018 and 2030. Our most conservative scenario analysis produces a lower-bound estimate of 209 million additional school days while the upper-bound estimate is approximately 836 million school days.

***School days by girls in agricultural households.*** If the analysis of lost school days is limited to only girls, the number of additional school days from 2018 to 2030 would be approximately 235 million school days.

***Caregiving days by women in agricultural households.*** With our base case scenario analysis, the number of caregiving days by women for children with malaria would be reduced by approximately 411 million days. The lower bound for this estimate is 73 million caregiving days while the upper bound is 1.1 billion days.

### 2018 to 2040: Impact of Malaria Elimination Path on SDGs

In Table 5, we present the results of our analysis of how eliminating malaria would affect our SDG indicators from 2018 through 2040.

**Table 5.** Impact of malaria elimination path on Sustainable Development Goals (2018 to 2040).

		Most Conservative Scenario (2018 to 2040)			Base Case Scenario (2018 to 2040)			Least Conservative Scenario (2018 to 2040)		
Sustainable Development Goal	Indicator	Status Quo	Elimination Path	Impact by 2040	Status Quo	Elimination Path	Impact by 2040	Status Quo	Elimination Path	Impact by 2040
#1: No Poverty	# of lost work days from malaria cases among agric. HHs	1,807,382,830	903,691,415	-903,691,415	6,099,917,052	3,049,958,526	-3,049,958,526	12,651,679,811	6,325,839,905	-6,325,839,905
#3: Good Health & Well-being	# of malaria cases among agric. HHs	1,129,614,269	564,807,134	-564,807,134	1,694,421,403	847,210,702	-847,210,702	2,259,228,538	1,129,614,269	-1,129,614,269
#4: Quality Education	# of lost school days by children due to malaria cases among agric. HHs	1,355,537,123	677,768,561	-677,768,561	3,049,958,526	1,524,979,263	-1,524,979,263	5,422,148,490	2,711,074,245	-2,711,074,245
#5: Gender equality	# of lost school days by girls due to malaria cases among agric. HHs	677,768,561	338,884,281	-338,884,281	1,524,979,263	762,489,631	-762,489,631	2,711,074,245	1,355,537,123	-1,355,537,123
#5: Gender equality	# of caregiving days by women due to malaria cases among agric. HHs	474,437,993	237,218,996	-237,218,996	2,668,713,710	1,334,356,855	-1,334,356,855	7,116,569,894	3,558,284,947	-3,558,284,947

***Malaria cases among agricultural households.*** Approximately 847 million malaria cases would be prevented among agricultural households from 2018 through 2040 according to our base case scenario analysis. The lower bound of this estimate of the number malaria cases prevented is 565 million, while the upper bound estimate is approximately 1.1 billion.

***Work days among agricultural households.*** According to our base case analysis, approximately 3 billion additional work days among agricultural households would be gained if malaria were eliminated by 2040 relative to the Status Quo Path. Our most conservative scenario analysis generates an estimate of 904 million work days prevented for the lower bound and our least conservative scenario estimates the number of work days prevented as 6.3 billion.

***School days among children in agricultural households.*** Approximately 1.5 billion school days would be gained from 2018 through 2040 by achieving malaria elimination in 2040. The lower bound estimate for this analysis is 678 million school days while the upper bound estimate is 2.7 billion school days.

***School days by girls in agricultural households.*** The number of school days gained for girls from 2018 through 2040 would be approximately 762 million, with a lower bound estimate of 339 million school days and an upper bound estimate of 1.4 billion school days.

***Caregiving days by women in agricultural households.*** According to our base case analysis, the number of caregiving days by women would be reduced by 1.3 billion days from 2018 to 2040. Our most conservative scenario generates a lower bound estimate of 237 million caregiving days, while the upper-bound estimate is a decrease of approximately 3.6 billion caregiving days.

## Discussion

To our knowledge, we have developed the first estimates of the number of malaria cases among agricultural households in sub-Saharan Africa and the potential impact of suppressing malaria among these households on four indicators related to the SDGs.

We would expect that our estimates of the impact of suppressing malaria on the selected indicators for the SDGs are conservative. A larger number of agricultural households in sub-Saharan Africa in 2018 and a larger number of malaria cases among those households in that year would result in a larger impact on the malaria elimination indicators for these households. As indicated in the Methods section, our estimates for the number of agricultural households in sub-Saharan Africa are conservative, which would imply that our estimates of the impact of suppressing malaria among these households on the indicators for the SDGs are also conservative.

### Topics for future research

One of the objectives of this study was to identify where additional research is needed to develop better estimates of how eradicating malaria by 2040 would affect agricultural households. The results of this study are meant to be a starting point for encouraging researchers to address research questions appropriate for the goal of eradicating malaria, allowing research to move away from the research questions and methodologies common in the 1990s and early 2000s when the goal was to simply “control” malaria.

This study examined how suppressing malaria over a twenty-one-year period, with the goal of malaria eradication by 2040, would affect approximately 324 million people living in agricultural households throughout 35 countries in sub-Saharan Africa. This is an ambitious research question to address in a single study. However, this research question is an example of the new lines of research that are needed to generate evidence that would help our understanding of how eradicating malaria would affect individuals in these regions. If the goal is to eradicate malaria by 2040, it is important to understand the cost and benefit of achieving that goal. Agricultural households in malarious regions of sub-Saharan Africa experience high rates of malaria transmission and high levels of poverty. Most studies of malaria’s impact on these households have used data from one community that were collected over less than two years. One rationale for this type of study is that a researcher can collect higher quality data in one community over a short period of time and, therefore, develop more rigorous estimates of malaria’s impact on agricultural households in that community over that period. While these studies may have been informative when the goal was to “control” malaria, they are not sufficient if the goal is to *eradicate* malaria.

One potential criticism of this study is that the quality of the data we used varies. While we would agree that there are differences in data quality across countries, this should not be an excuse for not addressing the issues covered in this study. To avoid this research question due to concerns about data quality would be to say, in effect, that researchers cannot quantify how eradicating malaria would affect more than 300 million people in regions with some of the highest levels of malaria transmission in the world. 

Higher quality data would of course facilitate more rigorous estimates regarding the consequences of eradicating malaria on these households. Our analysis identified two types of data which should be a priority for countries and donors. First, higher quality sub-national data are needed for the number of agricultural households throughout each of the 35 countries included in this analysis. Second, longitudinal data should be collected from agricultural households to examine how the malaria burden, household decisions and household income would change over time if malaria was suppressed.

Multiple methodologies could be used to examine how eradicating malaria by 2040 would affect agricultural households in sub-Saharan Africa. The methodology used in this study is meant to be a starting point for developing more complex future methodologies. One aspect of our methodology that could be strengthened with additional research is how the parameters used in our model would be affected over time as acquired immunity levels change. We would expect that the suppression of malaria over the next two decades would shift the malaria morbidity burden to older age groups and increase the number of work days lost per adult malaria case.

### Policy implications

Our study represents the first attempt to quantify the impact that malaria eradication would have on agricultural households across sub-Saharan Africa in terms of work days missed, school days missed and caregiving days provided. Achieving malaria eradication by 2040, and the resulting positive effect on the SDGs, will require that vector control interventions continue to play a key role in anti-malaria programs. Vector control has been shown to be the most effective intervention for reducing the malaria burden ( Bhatt *et al*., 2015). Long-lasting insecticide-treated bed nets and indoor residual spraying were responsible for 78% of the reduction in malaria cases between 2000 and 2015 ( Bhatt *et al*., 2015). Most successful vector control interventions use insecticides that have been repurposed from modern agriculture. The impact of these interventions, however, is being compromised by the development of resistance to the classes of insecticides currently used in vector control.

Investments by agricultural chemical companies in developing and delivering novel vector control tools will likely be essential to achieving malaria eradication by 2040. These investments will not only play a key role in improving the health of agricultural households in Africa but could also significantly accelerate growth in the incomes of these farmers by increasing their productivity.

To better understand how investments by agricultural chemical companies in vector control tools would affect agricultural households over the next 20 years, additional research is needed in three areas. First, researchers need to examine how suppressing malaria risk among farmers in Africa would affect their decisions of which crops to plant. Second, analyses are needed of the relationship between an increase in the income of agricultural households and any change in their malaria risk. There is some evidence that higher incomes among agricultural households lead to reduced malaria risk ( Ijumba & Lindsay, 2001) and possibly an increased investment in housing ( Sachs, 2018), but additional research is needed. Finally, researchers should examine the relationship between higher incomes of farmers and their decisions regarding how much to invest in agricultural inputs.

## Data availability

The dataset for this research has been deposited in CSV format with the Harvard Dataverse repository: https://dx.doi.org/10.7910/DVN/ZFJ3XT ( Willis, 2018). Data are available under the terms of the Creative Commons Zero "No rights reserved" data waiver (CC0 1.0 Public domain dedication).
