# Supplementary material for: Eliminating malaria by 2040 among agricultural households in Africa: potential impact on health, labor productivity, education and gender equality
Source: Gates Open Res. 2018 Nov 9;2:33. Originally published 2018 Jul 19. [Version 2] doi: 10.12688/gatesopenres.12843.2 (PMC6266653; doi:10.12688/gatesopenres.12843.2)
Supplement: Peer review history

# Peer review: Reviewer response for version 2

**DOI:** 10.21956/gatesopenres.13966.r26747

**Published:** 2018-11-14

**Authors:** Joseph Njau

**Affiliations:** 1 School of Medicine, University of California, San Francisco, San Francisco, CA, USA

The revised version looks very good. The authors have done their best to address earlier concerns and given the data limitation in this area, I believe this is a ground breaking study that should challenge many malaria researchers to think outside the box in addressing these problems and hopefully find long term solutions to malaria problem in most of the low and middle income countries worldwide.

I have read this submission. I believe that I have an appropriate level of expertise to confirm that it is of an acceptable scientific standard.

# Peer review: Reviewer response for version 1

**DOI:** 10.21956/gatesopenres.13924.r26567

**Published:** 2018-07-31

**Authors:** Joseph Njau

**Affiliations:** 1 School of Medicine, University of California, San Francisco, San Francisco, CA, USA

The objective of this study was to examine how suppressing malaria among smallholder agricultural households in SSA over the next two decades will affect progress towards achieving sustainable Development Goals (SDGs). The study is innovative and uses new data source that is usually not often used in traditional malaria studies. The study also aims to break new grounds in malaria analyses by linking malaria elimination with achievement of UN SDGs.

 However, this study may not be as reproducible or generalizable because of the following limitations:

 1) Reliability of the data. In most cases, a lot of the data had to be imputed with a lot of assumptions made to achieve study goals.

 2) Some of the assumptions made are excessively broad and may be unrealistic. For instance, taking a single year malaria infection rate to project all future malaria infections until 2040 is unrealistic because we already do know that there are a lot of malaria infection fluctuations. We have seen low infections at the beginning of this decade, we have had high infection rates in the last two years because of increased rain intensities etc. So, it may not be appropriate to use single year estimates to project all future malaria infection rates until 2040

- I would suggest using other modeling/statistical approaches like Monte Carlo simulations based on up to five years of past malaria infection rates.
- Use PERT distribution to project different malaria infection rates over time and model the impact on malaria suppression based on such numbers.

3) The study may have been too ambitious in terms of number of countries included in the analysis. One could have started small with countries that had the most reliable data and get precise estimates from those countries first before expanding to include countries with less reliable data.

I have read this submission. I believe that I have an appropriate level of expertise to confirm that it is of an acceptable scientific standard, however I have significant reservations, as outlined above.

# Peer review: Reviewer response for version 1

**DOI:** 10.21956/gatesopenres.13924.r26577

**Published:** 2018-07-20

**Authors:** Abiodun Olusola Omotayo

**Affiliations:** 1 Faculty of Natural and Agricultural Sciences, North-West University (NWU), Potchefstroom, South Africa

The work has been identified to be a good research work towards a United Nation’s sustainable goals.

 The authors should have a second look at the work and revise accordingly by adding the suggested articles and look critically into the few comments identified.

 The authors should ensure the paper cite properly as there are still facts mentioned with no references.

 The work should go for technical English editing. Also, perfect the formatting to the dictates of the Journals authors’ guide. 

 The methodology is though not so robust but the beauty of the work has been properly showcased and articulated through the excellent scholarly discussion of the subject matter.

 Further comments can be found in an annotated copy of the article, accessed here.

I have read this submission. I believe that I have an appropriate level of expertise to confirm that it is of an acceptable scientific standard.
